# Inter-study reproducibility of cardiovascular magnetic resonance tagging

**DOI:** 10.1186/1532-429X-15-37

**Published:** 2013-05-10

**Authors:** Sirisha Donekal, Bharath Ambale-Venkatesh, Seth Berkowitz, Colin O Wu, Eui Young Choi, Veronica Fernandes, Raymond Yan, Ahmed A Harouni, David A Bluemke, Joao A C Lima

**Affiliations:** 1 Department of Cardiology, Johns Hopkins University, 600 N Wolfe Street, Baltimore, MD 21287, USA; 2 Department of statistics, National Institutes of Health, Two Rockledge Center, Bethesda, MD 20892, USA; 3 Radiology and Imaging Sciences, National Institutes of Health, 10 Center Drive, Bethesda, MD 20892-1074, USA

**Keywords:** CMR tagging, HARP, Test-retest reproducibility, SPAMM, Circumferential strain, Radial strain, Principal strains, Torsion

## Abstract

**Background:**

The aim of this study is to determine the test-retest reliability of the measurement of regional myocardial function by cardiovascular magnetic resonance (CMR) tagging using spatial modulation of magnetization.

**Methods:**

Twenty-five participants underwent CMR tagging twice over 12 ± 7 days. To assess the role of slice orientation on strain measurement, two healthy volunteers had a first exam, followed by image acquisition repeated with slices rotated ±15 degrees out of true short axis, followed by a second exam in the true short axis plane. To assess the role of slice location, two healthy volunteers had whole heart tagging. The harmonic phase (HARP) method was used to analyze the tagged images. Peak midwall circumferential strain (Ecc), radial strain (Err), Lambda 1, Lambda 2, and Angle α were determined in basal, mid and apical slices. LV torsion, systolic and early diastolic circumferential strain and torsion rates were also determined.

**Results:**

LV Ecc and torsion had excellent intra-, interobserver, and inter-study intra-class correlation coefficients (ICC range, 0.7 to 0.9). Err, Lambda 1, Lambda 2 and angle had excellent intra- and interobserver ICC than inter-study ICC. Angle had least inter-study reproducibility. Torsion rates had superior intra-, interobserver, and inter-study reproducibility to strain rates. The measurements of LV Ecc were comparable in all three slices with different short axis orientations (standard deviation of mean Ecc was 0.09, 0.18 and 0.16 at basal, mid and apical slices, respectively). The mean difference in LV Ecc between slices was more pronounced in most of the basal slices compared to the rest of the heart.

**Conclusions:**

Intraobserver and interobserver reproducibility of all strain and torsion parameters was excellent. Inter-study reproducibility of CMR tagging by SPAMM varied between different parameters as described in the results above and was superior for Ecc and LV torsion. The variation in LV Ecc measurement due to altered slice orientation is negligible compared to the variation due to slice location.

**Trial registration:**

This trial is registered as NCT00005487 at National Heart, Lung and Blood institute.

## Background

Cardiovascular magnetic resonance (CMR) can accurately and precisely quantify regional myocardial function, allowing early identification of regional dysfunction [1]. Measurement of global cardiac function does not take into consideration the incipient alterations of myocardial contractile behavior seen in several cardiovascular disorders [2]. Tissue Doppler imaging and speckle tracking are the echocardiographic techniques for assessment of regional myocardial function at a high temporal resolution. Tissue Doppler ultrasonography and strain imaging are widely available, but image acquisition is operator dependent and relies on geometric assumptions [3]. Speckle tracking echocardiography (STE) can be used to determine myocardial deformation, but this technique depends on image quality, cardiac rhythm, left ventricular size, and analysis software algorithm [4]. CMR offers different techniques for measuring regional myocardial function including myocardial tagging and phase contrast imaging [5,6]. Myocardial tagging remains the reference standard for assessment of regional myocardial function. Several techniques have been proposed and developed in CMR tagging: Harmonic Phase (HARP) imaging, displacement encoding with simulated echoes (DENSE) [7], and strain-encoded (SENC) [8] MR. HARP analysis is currently the most widely used method for quantitative analysis of tagged images since it is highly automated and thus limits both analysis time and subjective interference [9]. HARP facilitates the use of CMR tagging techniques in large-scale multicenter studies, such as MESA (Multi-Ethnic Study of Atherosclerosis) [10].

Several studies have shown the association between regional myocardial function and traditional cardiovascular (CV) risk factors and markers of subclinical CV disease [11-15]. Therefore, for CMR tagging to be robust and useful in clinical settings, we need to determine the reproducibility of strain measurements to compare strains across longitudinal studies. Good inter- and intra-observer agreement of quantitative regional function analysis using HARP has already been demonstrated [10]. To evaluate any physiological variation in strain measurement, we performed a repeat CMR tagging on a different day from the initial scan using the same image acquisition parameters. We also assessed the role of slice orientation and slice location on LV strain measurement. Our aim was to determine the inter-study reproducibility of CMR tagging by spatial modulation of magnetization (SPAMM) to quantify myocardial strain.

## Methods

### Study participants

MESA was a prospective, population-based, epidemiological study to investigate the prevalence and progression of subclinical cardiovascular disease in a multi-ethnic cohort (Caucasian, African American, Hispanic, and Chinese) of men and women 45 to 84 years of age. The characteristics of MESA subjects have been described previously [16]. CMR tagging was performed in a cohort of 1,030 from 6 different sites, out of which 330 participants were from Baltimore, Maryland. Of these 330 participants, 25 participants were available and consented to a repeat CMR tagging examination. The local ethics board committee approved the study. In this ancillary study, after obtaining informed consent, these 25 participants had CMR tagging performed twice over 12 ± 7 days (range, 7–28 days), 7 females (28%), 18 males (72%), mean age 66 ± 7.1 years, range 53–80 years, Caucasians 64%, and African Americans 36%.

### CMR

Myocardial CMR-tagged images were obtained with 1.5 T MR Systems (Avanto, Siemens Medical Solutions, Germany). Images were acquired using segmented k-space; electrocardiogram-gated fast low-angle shot (FLASH) pulse sequence. The average scan time was about three minutes. The parameters for tagged images included the following: field of view 360 × 360 mm, slice thickness 10 mm, slice gap 10 mm, Echo time 2.5 ms, flip angle 10°, matrix size 256 × 128, phase-encoding views per segment 4 to 9, spatial resolution 1.4 × 2.8 × 10, temporal resolution 35 ms, tag spacing 7 mm.

The technicians were trained on the MESA CMR protocol and appropriate instructions were provided for the tagging sequences. Images were acquired at resting lung volume and the basal slice was chosen 2 cm below the mitral valve. The short axis images were obtained perpendicular to the inter-ventricular septum, with the three slices planned at the systolic phase. To evaluate any physiological variation in strain measurement, participants were advised to return on a different day for a repeat scan, with no restrictions to diet prior to the scan. The purpose of obtaining the study on a different day—within a period of no expected clinical change in the participant—from the initial scan was also to ensure that the strain measurements could be reliably obtained in the LV if the tagging protocol for appropriate short axis plane placement was followed consistently by the technician. This was to approximate to a clinical practice scenario where the sequential scans were performed by different technicians.

**Figure 1:**
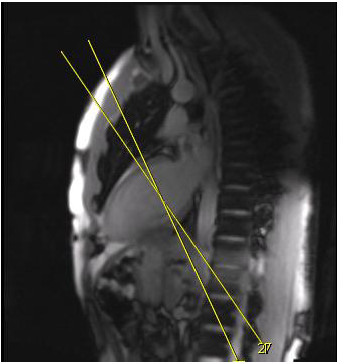
**Plane of short-axis to assess the role of slice orientation on LV Ecc measurement.** After obtaining initial tagged images acquisition was repeated with slices rotated ± 15° out of true short-axis.

To assess the role of slice orientation on LV Ecc measurement, two healthy volunteers were enrolled. After obtaining the initial scan, the image acquisition was repeated with slices rotated ±15 degrees out of the true short axis, followed by a repeat scan in true short axis. The volunteer was then advised to have a second scan in the true short axis plane 15 minutes from completion of the initial scan (Figure 1). To assess the role of slice location on LV Ecc measurement, two healthy volunteers had whole heart tagging with multiple short axis-tagged slices obtained from base to apex covering the entire heart. The parameters for tagged images were the same as described above with a slice gap of 10 mm and a slice thickness of 10 mm.

### Harmonic phase analysis

Tagged short-axis slices were analyzed by HARP (Diagnosoft, Inc., Palo Alto, CA). After importing the images, the short-axis images with horizontal and vertical tags were superimposed. The band-pass filter was selected automatically by HARP on the spectral peak adjacent to the Fourier space to produce the harmonic images. Endocardial and epicardial contours were manually traced on the image in end-systolic phase in each slice. HARP then automatically segmented the LV myocardium in 24 equal-sized regions each, with three layers: subepicardium, midwall, and subendocardium. This was visualized as a circular grid and tracked along the remaining cardiac phases. A few interactive corrections of the contour tracking were performed as necessary to obtain satisfactory matching. The average analysis time per study was about 10 minutes with a range of 8–12 minutes. To assess intraobserver reproducibility, a single reader performed the myocardial strain analysis twice for all image data sets. To assess interobserver reproducibility, a second reader performed analysis of all the data sets.

Peak midwall systolic strain (peak segmental strain) was determined for 16 segments of the AHA 16-segment model [17] using MATLAB software (The Math Works, MA, USA) [10]. Average midwall strain was calculated for each of the three slices averaging the corresponding peak segmental strain values from the AHA 16-segment model. The strain parameters obtained include Circumferential shortening, Ecc; radial thickening, Err; maximal elongation, Lambda 1; maximal shortening, Lambda 2; angle between the direction of Ecc, and Lambda 2, α (angle from here after). Strain rates were obtained by taking the first derivative of circumferential strain measurements over time for each LV segment. The time to peak systolic circumferential strain (ms), systolic circumferential strain rate (1/s), and early diastolic circumferential strain rate (ratio/s) were measured for the mid-ventricular midwall. Torsion has been defined in different ways in the literature [18]. We used the definition of LV Torsion as the difference in rotation (ϕ) between base and apex divided by the distance (D) between the measured locations of base and apex. Time to peak torsion, systolic torsion rate, and early diastolic torsion rate were determined.

### Statistical analysis

All continuous variables were represented by mean ± standard deviation and Categorical data were presented as percentages. Paired *t*-test was used to determine the differences in continuous variables. All tests were two-tailed and a *p* value <0.05 was considered to be statistically significant. The difference in strain variables between two exams was represented by mean difference and standard deviation of mean difference. Reliability was assessed using the intraclass correlation coefficient (ICC) with a model of absolute agreement; absolute measurement error was estimated by the standard error of measurement (SEM) and smallest detectable change (SDC) [19]. The SEM—defined as SEM = SD x √ (1-ICC) where SD = standard deviation of mean difference—takes the amount of measurement error into consideration and quantifies the within-subject variability. SDC—calculated as SDC = 1.96 x SEM x √2, where 1.96 corresponds to 95% confidence interval and the square root of 2 is to adjust for sampling from two different measurements—represents the 95% confidence that a change in the measurement exceeding this threshold is true and reliable and not just a measurement error. Bland-Altman analysis and Passing-Bablok regression [20] was performed to visualize the agreement and measurement error between the repeated studies. The degree of agreement between the two studies was determined by the mean difference and 95% confidence intervals of mean difference [21]. Statistical analysis was performed using SPSS statistical software version 19 (SPSS Inc., Chicago). Bland-Altman analysis and Passing-Bablok regression was performed using MedCalc, version 10.2.0.0 (MedCalc Software, Mariakerke, Belgium).

## Results

### Participant details

The mean age of these 25 participants was 66.4 ± 7.15 years (18 men and 7 women). Of these, 28% had diabetes mellitus, 56% were hypertensive, 64% were current smokers, and 16% had hyperlipidemia. There was no significant difference between the heart rates at both exams (61.6 ± 14.8 at exam 1, 62.7 ± 16.3 at exam 2 with a *p* value of 0.81). Systolic and diastolic blood pressure (SBP, DBP) was similar in both exams (SBP = 122.3 ± 18.1 and 119.5 ± 14.3 with a *p* value of 0.4; DBP = 72.5 ± 9.8 and 71.8 ± 8.7 with a *p* value of 0.6 at exam 1 and exam 2 respectively). Image quality was good for analysis in all the subjects. Multivariable linear regression demonstrated that traditional risk factors (age, gender, ethnicity, heart rate, and systolic and diastolic blood pressure) had no significant association on the reproducibility of strain measurements.

### Intraobserver and interobserver reproducibility

Intra- and interobserver reproducibility of strain and torsion parameters is demonstrated in Table 1. Intra- and interobserver reproducibility was excellent for average midwall peak Ecc and Lambda 2, LV torsion and peak diastolic torsion rate (ICC range 0.7 to 0.9). Err, Lambda 1 and angle had excellent intra- and interobserver reproducibility in the basal and mid slices compared to apical slices. Time to peak Ecc had moderate intraobserver reproducibility (ICC = 0.5) compared to interobserver reproducibility (ICC = 0.3). Time to peak torsion had excellent intraobserver reproducibility (ICC = 0.9) than interobserver agreement (ICC = 0.5), while peak systolic torsion rate had moderate interobserver reproducibility (ICC = 0.6) compared to intraobserver reproducibility (ICC = 0.4). Circumferential systolic and early diastolic strain rates were less reproducible compared to Ecc.

**Table 1:** Intraobserver and Interobserver reproducibility of LV strain and torsion

	**Slice**	**Intraobserver ICC**	**Interobserver ICC**
Ecc	Base	0.8**	0.8**
	Mid	0.77**	0.7 **
	Apex	0.8**	0.8 *
Err	Base	0.9**	0.9 **
	Mid	0.9**	0.8 **
	Apex	0.5**	0.7 **
Lambda 1	Base	0.8**	0.8 **
	Mid	0.8**	0.6 **
	Apex	0.6**	0.7 **
Lambda 2	Base	0.9**	0.8 **
	Mid	0.7**	0.7 **
	Apex	0.8**	0.8 **
Angle	Base	0.7**	0.7 **
	Mid	0.7**	0.5 **
	Apex	0.5*	0.5 *
Time to peak Ecc	Mid	0.5*	0.3 *
Systolic circumferential strain rate	Mid	−0.1	0.4*
Early diastolic circumferential strain rate	Mid	0.5*	0.2
Torsion	-	0.9**	0.9 **
Time to peak torsion	-	0.9**	0.5 *
Peak systolic torsion rate	-	0.4*	0.6 *
Peak diastolic torsion rate	-	0.8**	0.7 **

*Ecc* Circumferential strain, *Err* Radial strain.

ICC = Intraclass correlation coefficient.

** = *p* <0.0001.

* = *p* <0.05.

### Inter-study reproducibility

#### Strain and strain rates

**Figure 2:**
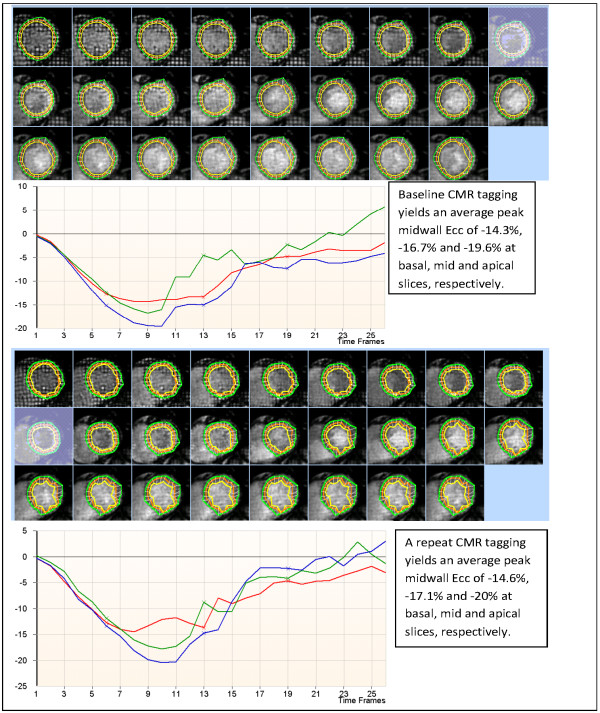
**CMR tagging images of a participant obtained 3 weeks apart.** Top: Initial scan; Bottom: Repeat scan.

The average peak midwall Ecc, Err, Lambda 1, Lambda 2, and angle α in the basal, mid and apical slices were similar in both exams (*p* >0.05) (Table 2). All strain parameters had superior intra- and interobserver reproducibility compared to inter-study reproducibility. The inter-study reproducibility of strain measurements was variable. Figure 2 displays the images of tagged short-axis slices from the initial and repeat scans of a participant, and the corresponding Ecc strain curves. ICC, SEM, and SDC of all strain variables are shown in Table 2.

**Table 2:** Inter-study reproducibility of LV strain and strain rates

**LV parameter**	**Slice**	**Exam 1**	**Exam 2**	**Mean difference (SD)**	**ICC**	**SEM**	**SDC**
Ecc, %	Base	−14.2	−13.9	−0.2 (1.6)	0.74**	0.8	2.3
	Mid	−14.7	−15.2	0.5 (1.9)	0.73**	1.0	2.7
	Apex	−15.6	−15.01	−0.6 (1.2)	0.89**	0.4	1.1
Err, %	Base	18.4	19.1	−0.7(5.3)	0.2	4.7	13.1
	Mid	16.6	17.1	0.5 (4.1)	0.58*	2.7	7.4
	Apex	15.1	13.9	1.2 (6.8)	0.3	5.7	15.8
Lambda 1, %	Base	23.1	24.0	−1.1 (5.2)	0.4*	4.0	11.2
	Mid	22.8	23.1	0.3 (5.2)	0.6**	3.3	9.1
	Apex	21.2	23.2	2.04 (7.2)	0.47*	5.2	14.5
Lambda 2, %	Base	−16.1	−16.6	0.5 (1.9)	0.66**	1.1	3.1
	Mid	−17.2	−17.6	0.4 (2.3)	0.5*	1.6	4.5
	Apex	−18.7	−19.4	0.7 (2.8)	0.47*	2.0	5.6
Angle, α	Base	11.4	10.0	1.3 (4.8)	0.05	4.7	13.0
	Mid	13.3	12.1	1.1 (5.6)	0.05	5.5	15.1
	Apex	16.2	17.2	−0.9 (6.6)	0.06	6.4	17.7
Time to peak Ecc (ms)	Mid	295.8	306	−10.4 (81)	0.4 *	62.7	173.8
SSR (1/s)	Mid	−79.6	−85.1	5.4 (15.7)	0.58*	10.2	28.2
EDSR (1/s)	Mid	72.2	70.8	1.4 (43.7)	0.35*	35.2	97.6

*SSR* systolic circumferential strain rate, *EDSR* Early diastolic circumferential strain rate.

*SD* standard deviation of mean difference, *ICC* Intra-class correlation coefficient, *SEM* standard error of measurement, *SDC* Smallest detectable change.

** = *p* <0.0001, * = *p* <0.05.

**Figure 3:**
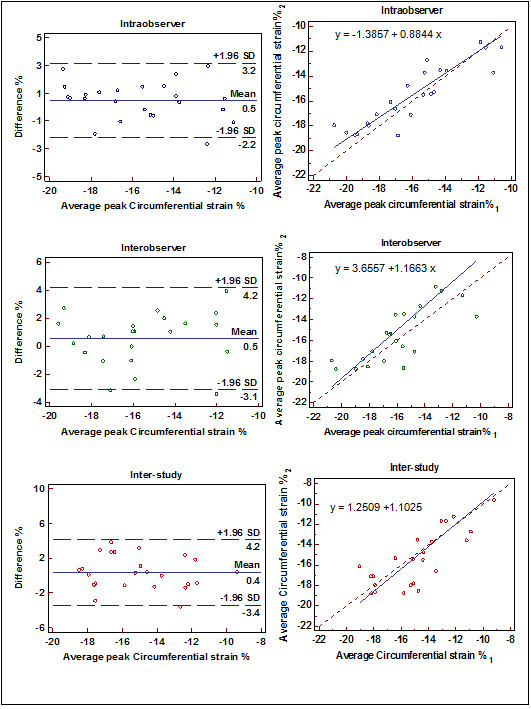
Intraobserver (top), interobserver (middle) and inter-study (bottom) reproducibility of average peak Ecc at mid ventricle: Bland-Altman plot (left) and Passing-Bablok regression (right) of mid-ventricle SD = Standard deviation.

Ecc had excellent inter-study reproducibility in the basal, mid, and apical slices (ICC = 0.74, 0.73 and 0.89; SDC = 2.3, 2.7 and 1.1, respectively) (Table 2). Intra-, interobserver, and inter-study reproducibility of mid-ventricular midwall average peak Ecc is demonstrated by Bland-Altman and regression plots in Figure 3. Lambda 1 and Lambda 2 had moderate inter-study reproducibility (ICC = 0.4, 0.6 and 0.47; SDC = 11.2, 9.1 and 14.5 for Lambda 1 while ICC = 0.66, 0.5, and 0.47; SDC = 3.1, 4.5 and 5.6 for Lambda 2) in the basal, mid and apical slices, respectively. Err had moderate inter-study reproducibility in the mid slice (ICC = 0.58, SDC = 7.4); however, Err was not reproducible in basal and apical slices. Angle was the least reproducible strain measurement (ICC = 0.05, 0.05 and 0.06; SDC = 13, 15.1 and 17.7 at basal, mid and apical slices, respectively).

Time to peak strain and strain rates at the mid ventricle are represented in Table 2. Time to peak Ecc, circumferential systolic, and early diastolic strain rate were similar between the exams (*p* >0.05). Circumferential systolic strain rate was moderately reproducible between the exams (ICC = 0.58, SDC = 44), while time to peak Ecc and early diastolic strain rate were poorly reproducible (ICC = 0.4 and 0.3; SDC = 173.8 and 28.2 respectively)

#### Torsion and torsion rates

**Figure 4:**
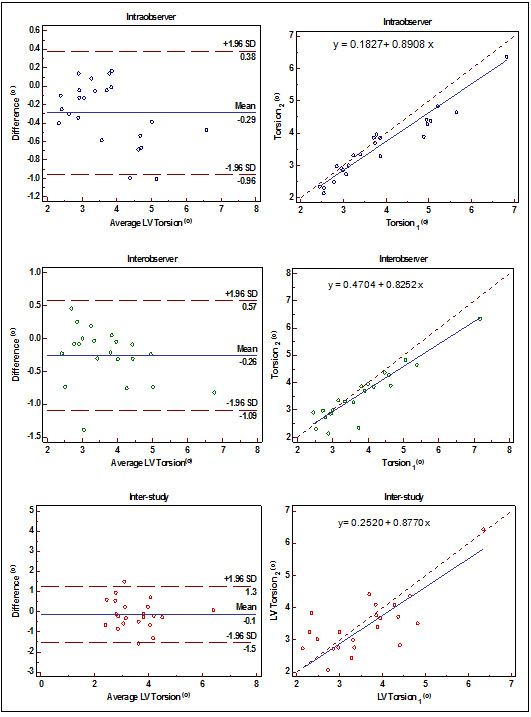
Intraobserver, interobserver and inter study variability of Torsion: Bland Altman plot (left) and Passing-Bablok regression (right), SD = Standard deviation.

LV torsion was similar between the two exams (*p* >0.05) and had excellent inter-study reproducibility (ICC = 0.73, SDC = 1.1). Intra-, interobserver, and inter-study reproducibility of LV torsion are displayed by Bland-Altman and regression plots in Figure 4. Time to peak torsion, peak systolic, and diastolic torsion rates were similar between the exams (*p* >0.05) (Table 3). Inter-study reproducibility of time to peak torsion was excellent (ICC = 0.77, SDC = 44); however, peak systolic and diastolic torsion rates were poorly reproducible between the exams (ICC = 0.4 and 0.37; SDC = 13.3 and 16.7, respectively).

**Table 3:** Inter-study reproducibility of LV Torsion and Torsion rates

**LV parameter**	**Exam 1**	**Exam 2**	**Mean difference (SD)**	**ICC**	**SEM**	**SDC**
Torsion °/cm	3.4	3.7	−0.3 (0.8)	0.73**	0.41	1.1
Time to peak Torsion (ms)	293.8	297.9	−4 (33.1)	0.77**	15.9	44.0
Peak systolic torsion rate ( °/s/cm)	16.1	18.2	−1.8 (6.2)	0.4 *	4.8	13.3
Peak diastolic torsion rate ( °/s/cm)	−17.2	−15.4	−1.8 (7.6)	0.37*	6.0	16.7

*SD* standard deviation of mean difference, *p* <0.05 is statistically significant, *ICC* Intra-class correlation coefficient, *SEM* standard error of measurement, *SDC* Smallest detectable change. ** = *p* value <0.0001, * = *p* value <0.05.

#### Role of LV slice orientation on LV Ecc measurement

**Figure 5:**
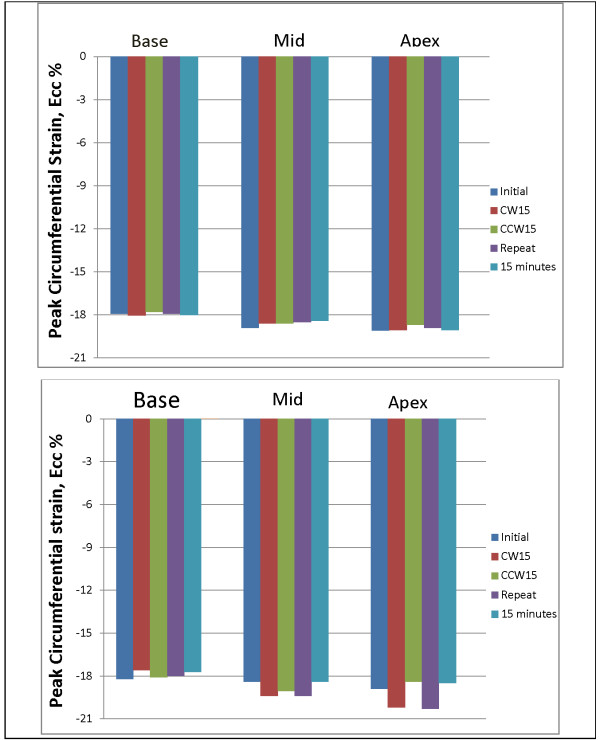
**LV Ecc after slice position alteration.** Initial: First scan, CW15 and CCW15: Clockwise and counterclockwise rotation of slices out of true short axis plane, Repeat: Repeat scan in true short-axis plane, 30 minutes: Volunteer moved out of the scanner, and was rescanned 15 minutes after completing the initial scan. Top: Volunteer 1 (healthy 29-yr-old male) Bottom: Volunteer 2 (healthy 25- yr-old female). Figure shows the peak LV Ecc in three slices with different image acquisitions.

Two healthy volunteers (29-year-old male and 25-year-old female) had CMR tagging with different slice orientations, as described in the methods section. The LV Ecc of these volunteers at basal, mid and apical slices is represented in Figure 5. The standard deviation of mean LV Ecc for these different image acquisitions was 0.09, 0.18, and 0.16 for volunteer 1, and 0.25, 0.5, and 0.9 for volunteer 2 at basal, mid, and apical slices, respectively.

#### Role of LV slice location on LV Ecc measurement

**Figure 6:**
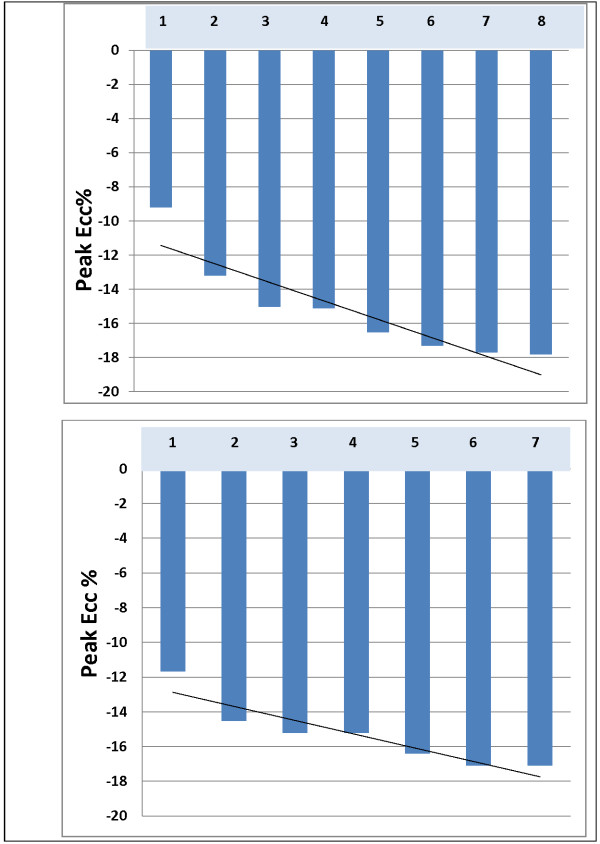
**Whole heart tagging.** Slice 1: Most basal; slice (7) 8: Most apical. Top: Volunteer 1 (44-yr-old healthy male) and Bottom: Volunteer 2 (42-yr-old healthy male) with whole heart tagging. As seen in the figure, LV Ecc progressively increases from base to apex and the difference in LV Ecc between the slices is more pronounced in most basal slices compared to the rest of the heart.

To evaluate the role of slice location, two healthy volunteers (42- and 44-year-old men) had whole-heart tagging with parallel short-axis 10 mm thick slices, 10 mm apart extending from base to apex. The average midwall peak LV Ecc progressively increased from base to apex (Figure 6). The mean difference in LV Ecc between the slices was more pronounced in most basal slices while the difference was minimal in the rest of the slices.

## Discussion

This study was conducted to evaluate the reproducibility of strain measurements obtained by CMR tagging. The results of the current study demonstrate that intra- and interobserver reproducibility of all LV strain and torsion parameters are excellent but intra- and interobserver reproducibility of strain rates and torsion rates is variable. As demonstrated by ICC, SEM and SDC, LV Ecc and torsion have excellent inter-study reproducibility while Lambda 1 and Lambda 2 have moderate inter-study reproducibility. Err is moderately reproducible in mid-ventricle and angle α is least reproducible. Time to peak Ecc, systolic and diastolic torsion are moderately reproducible between the studies. LV Torsion and time to peak LV torsion have excellent inter-study reproducibility, whereas systolic and diastolic torsion rates are moderately reproducible between the studies. Furthermore, the variation in LV strain measurement due to altered slice orientation is negligible compared to the variation due to slice location.

There are several technical factors that can affect the repeatability of a quantitative imaging technique. The technical factors involved in CMR tagging include strength of magnetic field, image acquisition, imaging orientation, slice location, and quality of tags. To avoid this variability, the studies were obtained at the same site and with the same scanner to eliminate the bias arising from the scanner. We used a standardized imaging protocol for choosing short-axis slices, but the technologists were blinded to previously chosen basal, mid, and apical slice positions on a 4-chamber localizer to approximate a clinical practice scenario, where sequential image acquisitions are often performed by different technicians. All imaging parameters were identical between exams, except for number of phases which is determined by heart rate.

In the current study, two-dimensional strains were calculated from three short-axis images. To evaluate whether shifting the plane of the short-axis slice would affect the estimated strain, the images were obtained with a 15 degree clockwise and counterclockwise rotation out of the true short axis, followed by a repeat image acquisition in the plane of true short axis. A second scan was performed 30 minutes after the initial scan. The resulting LV Ecc was similar to the different image acquisitions in all three LV slices, indicating that minimal alterations in slice orientation play a negligible role in the variability of LV Ecc measurement. Also, the results of whole heart tagging indicate that the variability of strain between the slices was linear from base to apex of the heart. The plots of average peak Ecc from each slice (Figure 6) demonstrate that, for every 5 mm slice gap, average peak Ecc increases by approximately 5 percent. This indicates that the variation in LV Ecc measurement can be introduced by slice position.

The variability can also be introduced by the image analysis software by horizontally and vertically tagged slice mis-registration, quality of mesh drawn, and filter-size adjustments. This variability was minimal as shown by the excellent intra- and interobserver reproducibility to measure regional myocardial deformation in the current study and in a previous study [10]. All the images were analyzed using the same protocol for analysis including image setup, adjusting the filter, drawing the mesh, and minimal manual correction of contours if needed.

Reproducibility of a quantitative measurement such as myocardial strain plays a key role in monitoring a patient’s response to therapeutic intervention. Understanding the measurement variability is also paramount to adequately define sample size in clinical trials so that aggregate results are clinically meaningful. Determining the inter-study variability of measurements is essential before using a technique for serial measurements. As demonstrated in the current study, the studies were repeated in a period of no expected clinical change and the resulting variability in the measurement of LV regional function appears to be because of inherent physiological variation in the strain itself, in addition to the variation because of slice position.

Several trends in observed variability can be explained by the physics of CMR tagging. Displacement and thus strain accuracy is directly related to the number of tags in a segment of interest. Due to myocardial geometry, there are more tags along a segment’s arc than along its radius [22]. Thus, Ecc is more reproducible than Err both in the present study and prior investigations of intra- and interobserver variability [10]. Similarly, Lambda 2—the more circumferential of the two principal strains—is more reproducible than Lambda 1 or the angle between. Similar to Ecc, torsion is calculated from circumferential displacement values, resulting in excellent reproducibility of LV torsion measurement.

Speckle tracking echocardiography (STE) has demonstrated similar results, with superior reproducibility of circumferential strain compared to radial strain, and superior reproducibility of peak strains compared to strain rates [23]. STE has been validated against sonomicrometry and tagged MRI [24], and has demonstrated that the intra- and interobserver reproducibility of strain parameters were superior to inter-study reproducibility as determined by speckle tracking echocardiography, but the reproducibility was superior to CMR tagging in our study, as demonstrated by higher ICC, lower SEM, and lower SDC for CMR tagging measured strain variables compared to STE [25]. Although good correlations have been demonstrated between strain measured by STE and CMR tagging, the strain measurements were systematically greater with STE than with CMR tagging [26]. STE currently has certain limitations such as moderate image quality and inter-vendor variability of LV strain measurement [27].

### Clinical implications

Several studies have been conducted in the MESA study using similar imaging protocol for CMR tagging; LV strain and torsion parameters were measured using HARP analysis. Previous studies results have demonstrated a significant association between LV strain and markers of atherosclerosis and subclinical cardiovascular disease [11-15]. Age-related changes in LV strain and torsion have been associated with ventricular and vascular remodeling which conferred a higher hazard of total cardiovascular events [28,29]. The excellent inter-study reproducibility of Ecc and LV torsion in the current study can thus aid in both diagnosis and follow-up of subclinical and clinical cardiovascular disease. In addition, the current study included both men and women, with a mean age of 66 years, who had underlying traditional cardiovascular risk factors, which is similar to a clinical setting. The image quality was adequate in all the participants; thus, the results from this study may be clinically applicable to the general population.

### Limitations

Our study used two-dimensional strain calculations to derive strain parameters; thus, the effect of through-plane motion was not determined. Several new techniques have been proposed for three-dimensional strain analysis including 3D complimentary SPAMM imaging [30], 3D HARP technique [31], zHARP [32], and phase unwrapping in HARP [33]. Larger studies using these newer techniques are needed to understand the regional myocardial function in different cardiovascular diseases. Another major limitation of this study is the image acquisition of only short-axis images and the lack of long-axis images, thus a lack of longitudinal strain measurement. Further studies are needed to determine the effect of scanner, magnetic field strength, and tagging pulse sequence on the reproducibility of strain measurement.

## Conclusions

In conclusion, our results demonstrate that CMR tagging by SPAMM yields excellent intra-, interobserver, and inter-study reproducibility of LV Ecc and torsion. In addition, Err, Lambda 1 and Lambda 2 have excellent intra- and interobserver reproducibility than inter-study reproducibility. The reproducibility of Ecc and torsion are superior compared to strain and torsion rates. The variation in LV Ecc measurement due to altered slice orientation is negligible compared to the variation due to slice location. The good reproducibility of this technique will enable CMR tagging to isolate meaningful trends in regional LV function in large cohorts of participants, such as the MESA study.

## Abbreviations

MESA: Multi-ethnic study of atherosclerosis; CMR: Cardiovascular magnetic resonance; SPAMM: Spatial modulation of magnetization; HARP: Harmonic phase analysis; LV: Left ventricle; Ecc: Circumferential strain; Err: Radial strain; ICC: Intra-class correlation coefficient; SEM: Standard error of measurement; SDC: Smallest detectable change.

## Competing interest

The authors declare that they have no competing interests.

## Authors’ contributions

SD: Study design, data analysis, data interpretation, manuscript drafting; SB: study design, data acquisition, manuscript revision; BA: data interpretation, manuscript revision; CW: study design, data interpretation, manuscript revision; EC: study design, manuscript revision; VF: study design, manuscript revision; RY: study design, manuscript revision; AE: study design, data acquisition, manuscript revision; DB: study design, manuscript revision; JL: principal investigator, study design, data interpretation, manuscript revision. All authors read and approved the final manuscript.
